# *Spir2*; a novel QTL on chromosome 4 contributes to susceptibility to pneumococcal infection in mice

**DOI:** 10.1186/1471-2164-14-242

**Published:** 2013-04-11

**Authors:** Laura Wisby, Vitor E Fernandes, Daniel R Neill, Aras Kadioglu, Peter W Andrew, Paul Denny

**Affiliations:** 1 MRC Mammalian Genetics Unit, Harwell, Oxon, OX11 0RD, UK; 2 Department of Infection, Immunity and Inflammation, University of Leicester, Leicester, UK; 3 Department of Clinical Infection Microbiology & Immunology, Institute of Infection & Global Health, University of Liverpool, Liverpool, UK

**Keywords:** Streptococcus pneumoniae, Host susceptibility, Host genetics, Quantitative trait loci, Model organism, Mouse, Bacterial infection, Inflammation

## Abstract

**Background:**

*Streptococcus pneumoniae* causes over one million deaths worldwide annually, despite recent developments in vaccine and antibiotic therapy. Host susceptibility to pneumococcal infection and disease is controlled by a combination of genetic and environmental influences, but current knowledge remains limited.

**Results:**

In order to identify novel host genetic variants as predictive risk factors or as potential targets for prophylaxis, we have looked for quantitative trait loci in a mouse model of invasive pneumococcal disease. We describe a novel locus, called *Streptococcus pneumoniae* infection resistance 2 (*Spir2)* on Chr4, which influences time to morbidity and the development of bacteraemia post-infection.

**Conclusions:**

The two quantitative trait loci we have identified (*Spir1* and *Spir2*) are linked significantly to both bacteraemia and survival time. This may mean that the principle cause of death, in our model of pneumonia, is bacteraemia and the downstream inflammatory effects it precipitates in the host.

## Background

*Streptococcus pneumoniae* is an important pathogen, responsible for causing pneumonia, bacterial meningitis, otitis media and sepsis, in humans. Pneumococcal disease causes a considerable burden on health services and is responsible, worldwide, for over 1.2 million deaths per year in children under the age of 5 years, with many of these cases occurring in developing countries [1].

New developments in conjugate vaccines are exciting but are based on the polysaccharide capsule of the pneumococcus and with more than 90 pneumococcal serotypes and genetic exchange of the capsular loci between *S. pneumoniae* contributing to the enhanced evasion from serotype specific antibody [2,3] alternative vaccines are still required. The drug of choice for treatment of pneumococcal infections has, for a long time, been penicillin. However over the last 30 years, resistance to penicillin and other antibiotics in *S. pneumoniae* has spread rapidly [4,5]. It is important to find new therapeutic targets to aid in the design and discovery of novel drugs as well as implementing genetic screening to identify individuals at risk so they can be targeted for prophylactic treatment.

Determination of genetic factors would open a radically new approach to prophylaxis and host defence. Studies of cause of death in adopted children and familial and twin studies have shown that susceptibility to infectious disease has a strong genetic component [6-9], but the genetics involved are complex and likely to be polygenic. The mapping of complex or quantitative trait loci (QTL) in naturally out-bred populations such as humans has been limited in success and identifying candidate genes has largely been restricted to association studies. Mouse models have been used to identify QTL with extensive homologies in humans and this approach has been successful in identifying infection susceptibility loci. One particularly successful example is the gene *Nramp1* that was identified as a candidate for susceptibility to tuberculosis by genome wide linkage studies in mice, and subsequently in humans by case control association studies [10,11].

We have reported previously on a mouse model of susceptibility to systemic pneumococcal infection in which BALB/c mice are resistant and CBA/Ca susceptible to intranasal infection with *S. pneumoniae* D39 [12]. A major QTL responsible in part for this difference in susceptibility has been mapped, in progeny of an F_2_ intercross, to proximal chromosome 7 and was named *Spir1* (*Streptococcus pneumoniae* infection resistance 1) [13]. Variants of several genes in the human population have been implicated in susceptibility to pneumococcal infection, including C reactive protein [14], Mannose binding lectin [15], TIRAP [16] and PTPN22 [17], but the mouse orthologs of these genes are not located in the *Spir1* locus [13].

In this present study, mapping of further progeny from the (BALB/cOlaHsd × CBA/CaOlaHsd)F_2_ (CCBAF_2_) intercross has identified a novel QTL on chromosome 4, which we have named *Spir2* (*Streptococcus pneumoniae* infection resistance 2). The contribution of this locus to susceptibility to pneumococcal infection was confirmed by congenic mapping.

## Results

### Identification of a novel QTL (*Spir2*) on chromosome 4 contributing to both survival time and bacteraemia

**Figure 1:**
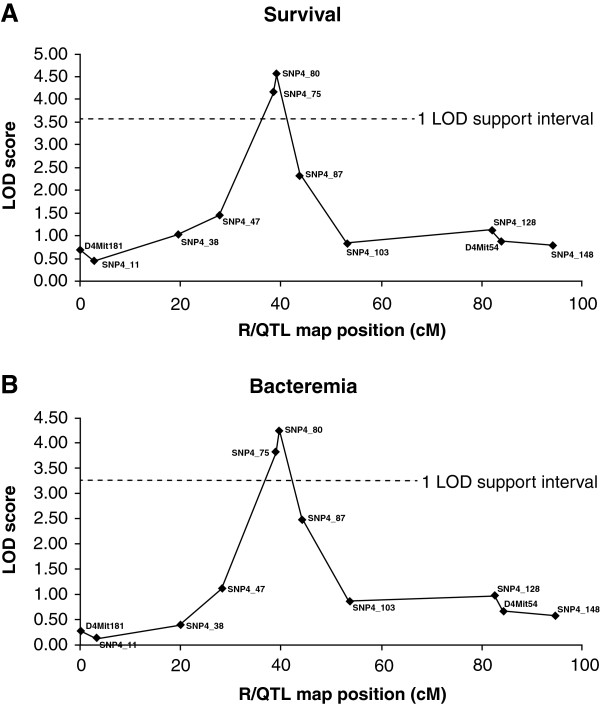
**LOD curves for chromosome 4 for the traits of survival (A) and bacteraemia (B).** The horizontal dotted line indicates the 1 LOD support interval. The peak of linkage for both traits was situated at SNP 4_80 (R/QTL analysis map position 39.2 cM, 82.5 Mb) with a LOD score of 4.56 for survival and 4.25 for bacteraemia.

Selective genotyping of CCBAF_2_ mice, with phenotypes representing the extremes of the response after infection challenge, identified a region of significant linkage on chromosome 4 with the traits of survival time and bacteraemia (Figure 1). The peak of linkage was located at SNP 4_80 (rs4224562) at 82.5 Mb. The QTL location on chromosome 4, estimated by the one LOD support interval, was from SNP 4_47 (rs13477699) at 50.7 Mb to SNP 4_87 (rs3022987) at 88.2 Mb and was ~ 38 Mb in size. The LOD score for the peak of linkage exceeded the genome wide significance threshold of 3.10 with a LOD score of 4.56 for survival and 4.25 for bacteraemia. The QTL identified on chromosome 4 was estimated to account for 8% of the phenotypic variance. Detection of the *Spir1* locus on chromosome 7 was replicated in this study, also accounting for ~ 8% of the phenotypic variance (data not shown).

### Effect of genotype at *Spir2* on phenotype

In the susceptible group of CCBAF_2_ mice the overall distribution of genotypes at SNP 4_80 were not significantly different from the expected Mendelian ratios. However, the distribution of genotypes in the resistant group of CCBAF_2_ mice was significantly different from the expected 1:2:1 ratio, at SNP 4_80, if genotype had no effect (χ^2^ = 7.2, df = 2, p = 0.027). There were a higher proportion of resistant mice heterozygous at this marker, compared to the proportion of resistant mice with CBA/Ca or BALB/c homozygosity at SNP 4_80 (Table 1).

**Table 1:** Numbers of CCBAF_2_ mice resistant and susceptible to pneumococcal infection, grouped by their genotype at SNP 4_80

**CCBAF2 genotype at SNP4_80**					
	CBA/Ca	Heterozygous	BALB/c	χ^2^	P value
**Observed**					
total	16	39	21	0.71	0.701
susceptible	12/16	12/39	14/21	5.37	0.068
resistant	4/16	27/39	7/21	7.21	0.027

Observed numbers of mice were compared to the expected numbers (1:2:1 ratio) if genotype had no effect using a χ^2^ test.

**Figure 2:**
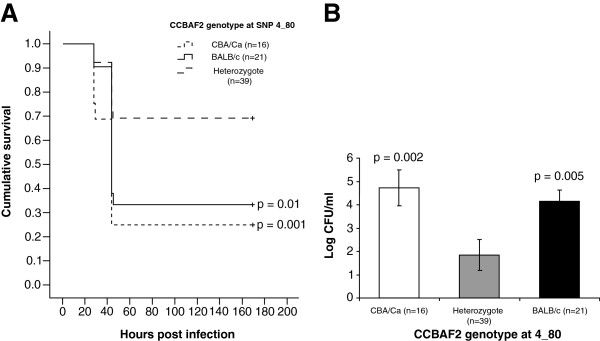
**Infection phenotypes for CCBAF2 mice heterozygous or homozygous for CBA/Ca or homozygous for BALB/c alleles at SNP 4_80.** (**A**) Kaplan-Meier survival curves, showing the cumulative survival (over time after intranasal infection with *S. pneumoniae*) for CCBAF_2_ mice heterozygous, homozygous for CBA/Ca or homozygous for BALB/c at SNP 4_80. Statistical significance (compared to heterozygote, pair-wise comparison with the Log Rank test) is indicated by p values on the graph. (**B**) Average numbers of *S. pneumoniae* (Log CFU/ml) in the blood at 24 hours post-infection for CCBAF_2_ mice grouped by their genotype at SNP 4_80. Error bars represent the standard error of the mean. The numbers of mice in each group are shown in brackets. Statistical significance (compared to heterozygote, unpaired *t*-test) is indicated by p values on the chart.

In order to investigate the effect of genotype at SNP 4_80 on the time to become moribund, a Kaplan-Meier survival analysis was performed (Figure 2A). CCBAF_2_ mice heterozygous for SNP 4_80 showed a significant difference in the time before exhibiting disease signs when compared to mice homozygous for BALB/c (p = 0.01) or CBA/Ca (p = 0.001). The mean and median survival times were calculated for each CCBAF_2_ genotype group. The median survival time, at which 50% of the group became severely lethargic, was 44 hours for both BALB/c and CBA/Ca genotypes. There was no median survival time calculated for the heterozygous genotype, as more than 50% of the group survived to the end of the experiment. The numbers of bacteria in the blood at 24 hours after intranasal infection were also significantly lower in CCBAF_2_ mice, which were heterozygous for the peak of linkage, when compared to those which were homozygous for BALB/c or CBA/Ca at the same SNP (Figure 2B). These data suggest that being heterozygous at this location on chromosome 4 is advantageous.

### Congenic breeding confirms the contribution of the *Spir2* locus to susceptibility to pneumococcal infection

The strategy of congenic breeding was used to replicate detection of the chromosome 4 QTL and to assess its contribution to the infection phenotype. Incipient congenic strains consisting of different portions of the chromosome 4 QTL from BALB/cByJ on a CBA/CaH background, and vice versa, were produced using a marker-assisted breeding scheme.

The percentages of heterozygosity observed in the N2 and N3 males used for breeding further generations were between 37 and 42% for the N2 generation and 7 to 10% for the N3 generation. Mice with the CBA/CaH *Spir2* locus on a BALB/cByJ background were bred to the N6 generation before intercrossing. These incipient congenics were named BYJCBAN6-4. Mice with the BALB/cByJ *Spir2* locus on a CBA/CaH background were bred to the N7 generation and were named CBABYJN7-4. The estimated percentage recipient genome in the final incipient congenic mice tested for infection susceptibility was 99.36 to 99.78%. Therefore these mice were similar to N9 mice produced by conventional congenic breeding.

### BALB/cByJ *Spir2* locus on CBA/CaH background (CBABYJN7-4)

A total of 75 CBABYJN7-4 intercross mice were tested for susceptibility to *S. pneumoniae* infection. Of these 75 mice 18 (24%) were resistant to pneumococcal infection and the remaining 57 (76%) were susceptible. Six of the susceptible mice survived 50 hours or longer, while the rest only survived to between 28 and 49 hours.

**Figure 3:**
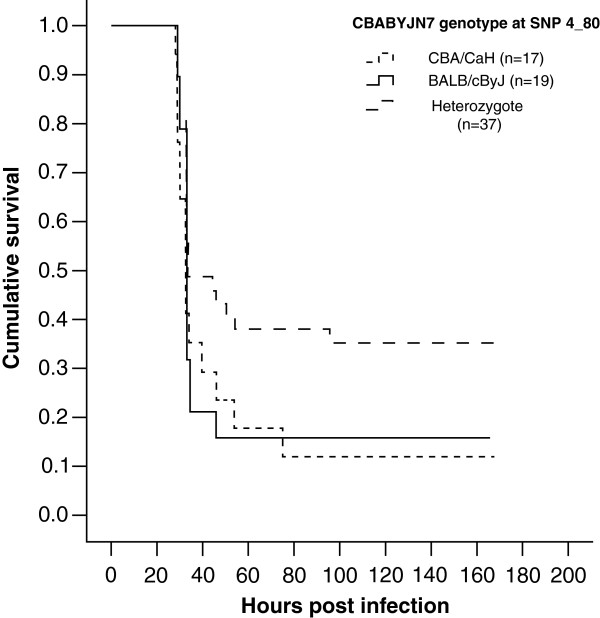
Kaplan-Meier survival curves, showing the cumulative survival for CBABYJN7-4 mice heterozygous, homozygous for CBA/CaH or homozygous for BALB/cByJ at SNP 4_80.

A survival analysis was performed on the CBABYJN7-4 mice for each SNP on Chr4 and it was found that animals that were heterozygous at SNP 4_80 survived longer by comparison with those homozygous for the CBA/Ca allele. Similar results were found at SNP 4_103 (Additional file 1: Table S2). These results do not achieve statistical significance (SNP 4_80 p = 0.064 and SNP 4_103 p = 0.087), yet are noteworthy because they show the same trend as the *Spir2* QTL detected in the CCBAF_2_. (Figure 3 and Additional file 2: Figure S1). There was no difference in time to morbidity between mice homozygous for BALB/cByJ and those homozygous for CBA/CaH at any Chr4 marker. There was also no significant difference in the numbers of bacteria in the blood at 24 hours post-infection in CBABYJN7-4 mice homozygous for BALB/cByJ or CBA/CaH or heterozygous for any of the chromosome 4 SNP markers (data not shown).

**Figure 4:**
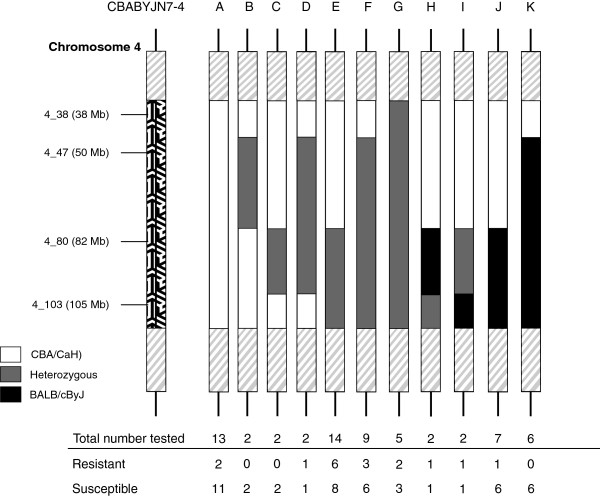
**Diagram representing chromosome 4 from the different CBABYJN7-4 haplotype groups named CBABYJN7-4-A to K.** The area spanning SNP markers 4_38 to 4_103 is highlighted with the positions of each the SNP markers used for genotyping shown on the left. Areas outside of these SNP markers (grey diagonal lines) are either homozygous for the recipient strain or heterozygous. Black represents regions homozygous for BALB/cByJ, white for regions homozygous for CBA/CaH and those in grey are heterozygous. The total number of mice from each group that were tested can be seen with the numbers of resistant and susceptible mice below.

**Figure 5:**
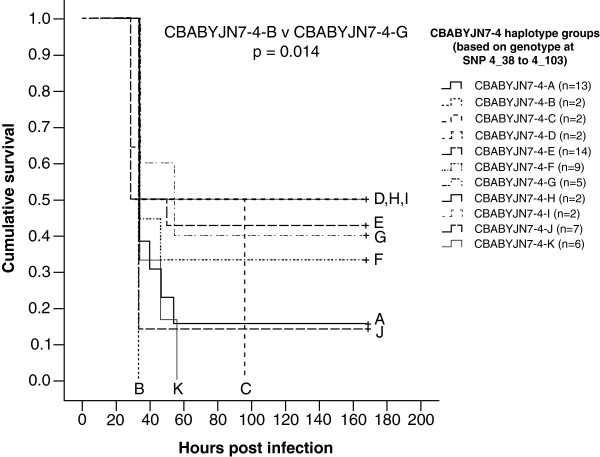
**Kaplan-Meier survival curves showing the cumulative survival for the CBABYJN7-4 haplotype groups.** The survival curve for each haplotype is labelled (**A** to **K**). Statistical significance (pair-wise comparison with the Log Rank test) is indicated by p values on the graph.

A total of 64 mice could be divided into 11 haplotypes based on their combination of genotypes at SNPs 4_38 to 4_103. These groups were called CBABYJN7-4-A to K (Figure 4). There was no significant difference in numbers of bacteria in the blood at 24 hours post-infection between any of the CBABYJN7-4 haplotypes (data not shown). However, there was a significant difference between the survival curves of CBABYJN7-4-B and G (p = 0.014) with a lower risk of disease in the CBABYJN7-4-G mice compared to the CBABYJN7-4-B mice (Figure 5).

### CBA/CaH *Spir2* locus on BALB/cByJ background (BYJCBAN6-4)

**Figure 6:**
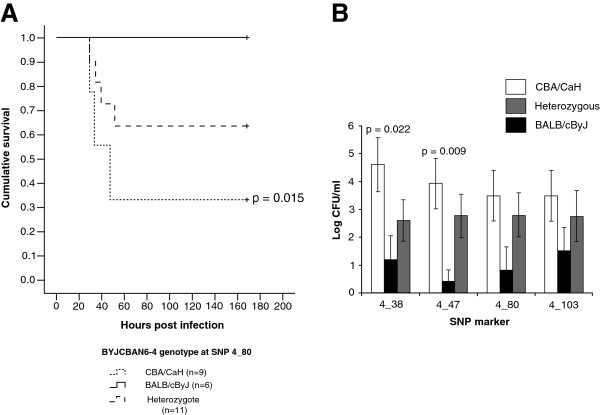
**Infection phenotypes for BYJCBAN6-4 mice. **Kaplan-Meier survival curves, showing the cumulative survival for BYJCBAN6-4 mice heterozygous, homozygous for CBA/CaH or homozygous for BALB/cByJ at SNP 4_80 (**A**). Statistical significance (compared to BYJCBAN6-4 mice homozygous for BALB/cByJ, pair-wise comparison with the Log Rank test) is indicated by p values on the graph. (**B**) Average numbers of *S. pneumoniae* (Log CFU/ml) in the blood at 24 hours post-infection for BYJCBAN6-4 mice grouped by their genotype at SNPs 4_38, 4_47, 4_80 or 4_103. Error bars represent the standard error of the mean. The numbers of mice in each group are shown in brackets. Statistical significance (compared to BALB/cByJ homozygosity, unpaired *t*-test) is indicated by p values on the graph.

A total of 26 BYJCBAN6-4 intercross mice were tested for susceptibility to infection with *S. pneumoniae*. Ten (38%) of these mice were susceptible and 16 (62%) were resistant to pneumococcal infection. An analysis of the survival of these mice, based on their genotype at markers on Chr4, revealed significant differences for SNPs 4_38, 4_80 and 4_103 (Figure 6A and Additional file 3: Figure S2). Mice homozygous for the BALB/cByJ allele of any of these three SNPS were at significantly less risk of disease than those homozygous for CBA/CaH at the same SNP. Survival curves for mice that were heterozygous for the SNP 4_38, were significantly different from those for CBA/CaH homozygotes (p = 0.007), with more heterozygotes surviving. There were no significant differences in the survival curves for animals of any of the genotypes for SNP 4_47, however this marker did follow the same trend as for SNPs 4_38, 4_80 and 4_103 (data not shown).

Numbers of bacteria in the blood of BYJCBAN6-4 mice were assessed at 24 hours post-infection. There were significantly more bacteria present in mice homozygous for the CBA/CaH allele at SNP marker 4_38 or 4_47 when compared with mice either heterozygous or homozygous for BALB/cByJ at the same SNP (p = 0.022 and 0.009 respectively). Although not significant, the trend was the same for SNP markers 4_80 and 4_103 (Figure 6B).

**Figure 7:**
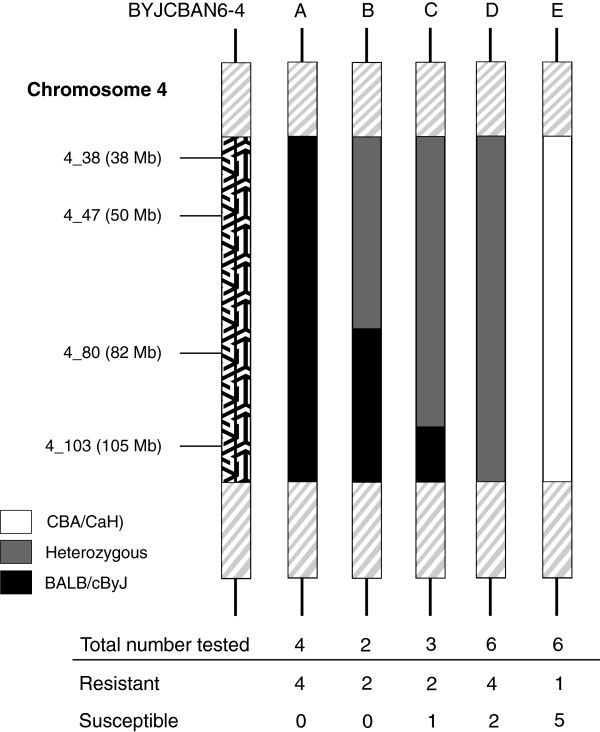
**Diagram representing chromosome 4 from the different BYJCBAN6-4 haplotype groups named BYJCBAN6-4-A to E.** The area spanning SNP markers 4_38 to 4_103 is highlighted with the positions of each the SNP markers used for genotyping shown on the left. Areas outside of these SNP markers (grey diagonal lines) are either homozygous for the recipient strain or heterozygous. Black represents regions homozygous for BALB/cByJ, white for regions homozygous for CBA/CaH and those in grey are heterozygous. The total number of mice from each group that were tested can be seen with the numbers of resistant and susceptible mice below.

**Figure 8:**
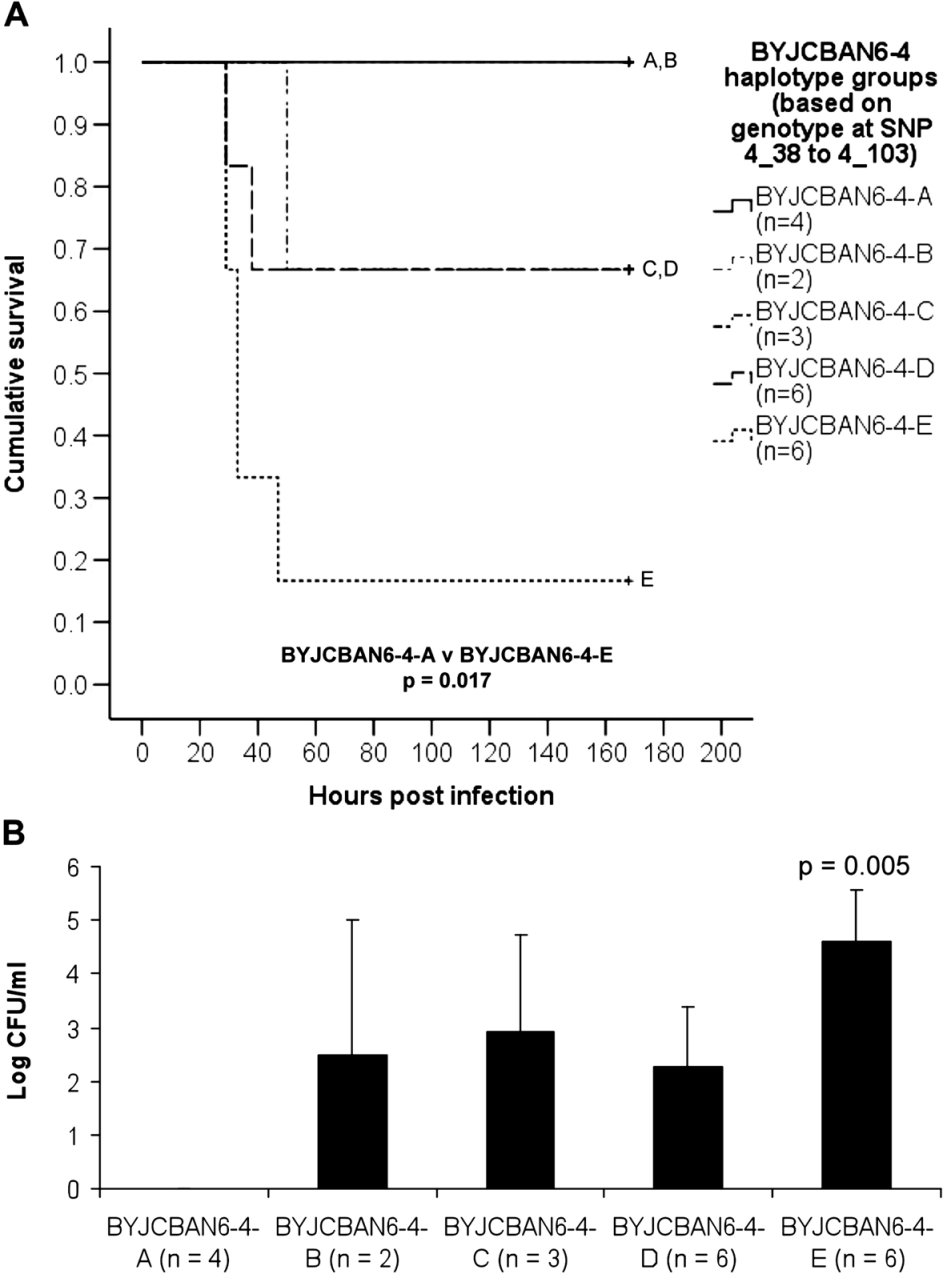
**Infection phenotypes for BYJCBAN6-4 mice with different haplotypes.** (**A**) Kaplan-Meier survival curves showing the cumulative survival for the BYJCBAN6-4 haplotypes. The survival curve of each group is labelled (A to E). Statistical significance (pair-wise comparison with the Log Rank test) is indicated by p values on the graph. (**B**) Average numbers of *S. pneumoniae* (Log CFU/ml) in the blood at 24 hours post-infection for each BYJCBAN6-4 haplotype. Error bars represent the standard error of the mean. The numbers of mice in each group are shown in brackets. Statistical significance (compared to BYJCBAN6-4-A, unpaired *t*-test) is indicated by p values on the graph.

Analysis of the combination of alleles at SNPs 4_38 to 4_103 was performed on the BYJCBAN6-4 mice. Of the 26 mice, 21 could be placed into one of five different haplotypes, named BYJCBAN6-4-A to E (Figure 7). Kaplan-Meier analysis of the five haplotype groups revealed a significant difference in the survival curve of BYJCBAN6-4-A mice, which were homozygous for BALB/cByJ at all four SNPs when compared with BYJCBAN6-4-E mice, which were homozygous for CBA/CaH at these SNPs (p = 0.017) (Figure 8A). Analysis of the bacteraemia results at 24 hours post-infection also revealed a significant difference between BYJCBAN6-4-A and E mice (p = 0.005). BYJCBAN6-4-E mice had significantly higher numbers of bacteria present in the blood when compared with BYJCBAN6-4-A mice, which had no bacteraemia at 24 hours (Figure 8B).

## Discussion

Selective genotyping of CCBAF_2_ mice from the phenotypic extremes was successful in identifying a novel QTL on chromosome 4 (named *Spir2*) contributing to susceptibility to pneumococcal disease. This type of selective genotyping has been implemented in various studies because it reduces laboratory costs and it is successful in gaining as much, if not more, information as genotyping the same number of random mice [18-21]. The *Spir2* peak of linkage was situated at SNP 4_80 and CCBAF2 mice that were heterozygous for this SNP had both significantly longer survival times and lower levels of bacteraemia, following intranasal infection, when compared with homozygous animals. Infection challenges of the Chr4 incipient congenic strains confirmed detection of the *Spir2* QTL observed in the CCBAF2 mice, but did not reduce the size of the critical region.

BYJCBAN6-4 mice, which had the *Spir2* region introgressed from CBA/CaH onto the BALB/cByJ background, had significantly shorter survival times after infection and significantly higher levels of bacteraemia at 24 hours when compared with mice heterozygous for the QTL or homozygous for BALB/cByJ. The effect of the QTL was different when on the CBA/CaH background. Interestingly, although not quite significant, mice heterozygous for SNPs 4_80 and 4_103 on the CBA/CaH background were less susceptible than those homozygous for CBA/CaH or BALB/cByJ. These results are similar to the effect observed in the analysis of the CCBAF2 QTL. Although the mechanisms are currently unclear, there have been reported cases in which heterozygous genotypes are advantageous over either homozygous genotype. A well-documented case of this phenomenon is the sickle cell gene haemoglobin (Hb) in humans. Carriers of the sickle cell trait are heterozygous for the Hb genotype (HbAS) and this heterozygosity seems to be protective against malaria, with lower mortality and parasitaemia when compared with either homozygous Hb genotypes (HbSS and HbAA) [22]. The differences observed in the phenotypes of the incipient congenics in our study were dependent on the recipient genome and this highlights the probable contribution of epistatic interaction effects from the background.

The region containing the *Spir2* locus is approximately 39 Mb in length and overlaps with several published QTL involved in immunity or susceptibility to infection; *Bbaa1* (*Borrelia burgdorferi* - associated arthritis 1), a locus contributing to severity of arthritis induced by *B. burgdorferi* infection [23], *Lprm1* (lymphoproliferation modifier 1), a QTL conferring susceptibility to autoimmune vasculitis [24], *Sle2* (systemic lupus erythmatosus susceptibility 2), contributing to B cell hyperactivity, an immunogenic phenotype caused by the autoimmune disease systemic lupus erythmatosus [25,26] and *Marif1* (macrophage-associated risk inflammatory factor 1) which affects inflammatory phenotypes of macrophages such as secretion of TNFα and IL-12p40 [27]. It is premature to speculate whether any of the genes underlying these QTLs are shared, but the *Marif1* locus is of interest because of the opposite Th1/Th2 bias of the inbred strains BALB/c and CBA/Ca [28] and because we [29] and others [30] have shown that macrophages play an important role in susceptibility to pneumococci.

The *Spir2* locus region contains 169 genes, according to Ensembl V.65. One strong candidate gene, based on it’s known role in regulating innate immunity, is *Tlr4*[31]*,* but non-synonymous differences in the sequence of *Tlr4* between BALB/cOlaHsd and CBA/CaOlaHsd were found to be common to other inbred strains, making it less likely to play a role in the *Spir2* locus (data not shown). It is clear that sequence analysis of the entire QTL would aid the prioritisation of other candidate disease genes for further study.

## Conclusions

The *Spir1* and *Spir2* loci are linked significantly to both bacteraemia and survival time ([13] and this work). This may mean that the principle cause of death, in our model of pneumonia, is bacteraemia and the downstream inflammatory effects it precipitates in the host.

## Methods

### Ethics statement

This study was performed in strict accordance with U.K. Home Office guidelines. Both the U.K. Home Office and the University of Leicester ethics committee approved the protocol. Every effort was made to minimize suffering and in bacterial infection experiments mice were humanely culled if they became lethargic. All animal experiments were carried out at the University of Leicester.

### Mice

BALB/cByJ and CBA/CaH were obtained from the MRC Mary Lyon Centre (MLC) in Harwell and were used for the congenic breeding scheme. The congenic breeding was performed at the MLC and infection studies were done at the University of Leicester.

### Congenic breeding scheme

A semi-speed congenic breeding scheme was implemented in this study. An alternating set of 73 SNP markers, spanning the genome, with an average spacing of 28 Mb, was used in order to reduce cost and time and ensure good coverage of the genome [32]. During the first two backcross generations (N2 and N3) the alternate genome scan was performed where at least 20 male progeny for each generation, which were heterozygous for the QTL, were typed genome wide in order to select the best male for breeding [32,33]. The next generations (from N4 to N6 or N7) were only genotyped for the chromosome 4 QTL markers. Once the mice reached the N6 or N7 generation, female mice heterozygous for the region of interest were crossed to males heterozygous for the same SNP markers to produce offspring for infection testing. Whole litters were tested for susceptibility to *S. pneumoniae*.

### Bacterial culture and infections

Incipient congenic mice were infected intranasally with *S. pneumoniae* D39 as described previously [12,13]. Blood was taken at 24 hours post-infection from the tail vein (for bacterial culture) and survival times of the mice were recorded. Mice that survived to the end point of the experiment (more than 168 hours) were considered resistant. All mice that succumbed to infection (the endpoint was severely lethargic) and had survival times less than 168 hours were considered susceptible.

### DNA extractions

For QTL mapping, DNA from the (BALB/cOlaHsd × CBA/CaOlaHsd)F_2_ (CCBAF_2_) intercross, reported in Denny et al., [13], was used. DNA samples from CCBAF_2_ mice from the phenotypic extremes were selected for QTL mapping. Thirty eight of the most susceptible mice (survival < 46 hours and high levels of bacteria in blood) and thirty eight of the most resistant mice (survival ≥ 168 hours and no bacteria in blood) were genotyped for linkage analysis. DNA was diluted to 5 ng/μl in double-distilled H_2_O.

In congenic breeding, DNA was extracted from ear clips using the Viagen DirectPCR ear lysis reagent (Viagen Biotech cat 402-E). 195 μl Direct PCR lysis reagent and 5 μl proteinase K (10 mg/ml) was added to each earclip and incubated overnight at 55°C. After digestion, the samples were heated to 85°C for 45 min and centrifuged for 10 seconds. 1 μl of lysate was used in each PCR reaction.

### SNP panel

The 76 CCBAF_2_ mice from the phenotypic extremes were genotyped by Pyrosequencing (as described below) across the whole genome using a panel of 73 SNPs with an average spacing of 28 Mb. Chromosome 4 was genotyped with an additional set of SNP markers to narrow down areas of suggestive linkage. A total of nine SNPs for chromosome 4 were typed by Pyrosequencing. Genotyping data from chromosome 4 microsatellite markers used in Denny et al., [13] were incorporated into the analysis. There were a total of 5 microsatellite markers that had been typed on 38 of the mice. Details of the SNP primers can be found in Additional file 4: Table S1.

### Primer design

SNPs were selected using the Mouse Phenome Database (http://phenome.jax.org/pub-cgi/phenome/mpdcgi?rtn=docs/home) and the SNP sequences were exported from the NCBI Entrez SNP database (http://www.ncbi.nlm.nih.gov/sites/entrez). Primers for Pyrosequencing were designed using the PSQ Assay Design software from Biotage AB. Primer sets of three primers for each SNP were designed, one pair of primers for the PCR (one of which was biotinylated) and a sequencing primer for the Pyrosequencing reaction. The minimum and maximum T_m_ for the PCR primers were 64 to 66°C. The sequencing primers were designed with a maximum distance of three bases from the SNP. Primers were manufactured by either Biomers.net or MWG-Biotech.

### PCR

10 μl PCR reactions were set up using 5 μl Qiagen Taq PCR master mix (cat. 201445), 0.2 μl forward primer and 0.2 μl reverse primer (at 10 pmol/μl), 2.6 - 3.6 μl nuclease free water and 1–2 μl DNA(~5 ng/μl). PCR reactions were run using the following PCR program: 95°C for 5 min, followed by 45 cycles of 95°C for 15 sec, 60°C for 30 sec and 72°C for 15 sec. The final extension step was for 5 min at 72°C.

### Pyrosequencing

10 μl PCR product, 2 μl streptavidin-Sepharose beads (GE Healthcare 17-5113-01), 38 μl binding buffer (Biotage AB 40–0033) and 30 μl H_2_O were combined in a 96-well plate and mixed vigorously on a plate shaker for 5 min so that the biotin-labelled PCR product bound to the streptavidin coated beads. The PCR products were then prepared using a vacuum work table (Biotage AB). The biotinylated PCR products, attached to the filter probes of the vacuum tool, were immersed in 70% (v/v) ethanol for 5 seconds, denatured in PyroMark Denaturation solution (Biotage AB 40–0034) for 5 sec (allowing only the biotin labelled strand of the PCR product to stay attached to the filter probes) and immersed in 1X PyroMark Wash buffer (Biotage AB 40–0035) for 5 sec. The single-stranded PCR products were then re-suspended in a PSQ HS 96-well plate containing 0.5 μl sequencing primer (at 10 pmol/μl) and 11.5 μl annealing buffer (Biotage AB 40–0036) per well.

The plate was incubated at 80°C for 2 minutes to allow the sequencing primer to anneal to the single-stranded PCR product. The PSQ 96-well plate and a PSQ HS 96 capillary dispensing tip holder (Biotage AB 60–0211) containing enzyme, substrate and dNTPs (PyroGold reagent kit Biotage AB 40–0047), were placed into a PSQ HS 96 Pyrosequencer (Biotage AB). The assays were performed and the data were analysed using the SNP software (Biotage AB).

### Linkage analysis

SNP genotype data from the 76 CCBAF_2_ mice and phenotype data from 168 non-genotyped CCBAF_2_ mice were incorporated into the linkage analysis. Data were analysed using R-QTL for non parametric analysis [34]. One thousand permutation tests were performed in order to establish genome-wide LOD significance thresholds at the 90% and 95% confidence level. Loci with LOD scores exceeding the 90% confidence level were identified as suggestive and those exceeding the 95% confidence level were identified as significant regions of linkage. The confidence interval for each QTL was determined by a one LOD support interval (−1 LOD drop from the peak of linkage). For the marker at the peak of each QTL, data were analysed using a Chi-squared (χ^2^) test. Numbers of mice resistant and susceptible to pneumococcal infection were grouped by their genotype at the appropriate SNP marker. Observed numbers of mice were compared to the expected numbers (1:2:1 ratio) if genotype had no effect (χ^2^). To investigate the effect the genotype, at the peak of linkage, on time to reach a moribund state, Kaplan Meier survival analysis was performed using the statistics package SPSS (version 17). The difference between the survival curves was analysed using the Log Rank test. p values of less than 0.05 were considered significant. The heritability equation (1-10^-2LOD/n^) was used to estimate the percentage of phenotypic variance accounted for by the QTL.

### Congenic breeding data analysis

Survival data and bacteraemia data were firstly analysed on a single SNP basis for chromosome 4. Mice were then grouped into shared haplotypes based on the combination of SNPs for chromosome 4 from SNP 4_38 to SNP 4_103. Survival data were analysed using Kaplan-Meier survival analysis with Log Rank pair wise comparison for statistical significance using SPSS (version 17). Bacteraemia data for the three genotypes (BALB/cByJ homozygous, CBA/CaH homozygous or heterozygous) were compared for each SNP using a two-tailed unpaired Student *t*-test. Numbers of bacteria in the blood in the haplotypes were compared in the same way. Results with p values less than 0.05 were considered significant.

## Abbreviations

df: Degrees of freedom; LOD: Logarithm of odds; QTL: Quantitative trait locus; SNP: Single nucleotide polymorphism; Spir2: *S. pneumoniae* infection resistance 2.

## Competing interests

The authors declare that they have no competing interests.

## Authors' contributions

LW performed the CCBAF_2_ and congenic SNP genotyping, management of the congenic breeding scheme, QTL and congenic mapping data analysis and interpretation of the results. DRN and VEF designed and performed PCR assays and sequencing. VEF performed the infection studies and bacterial culture. PWA, AK and PD conceived and designed the experiments. Overall project leadership was shared jointly by PW Andrew and Paul Denny. All authors read and approved the final manuscript.

## Supplementary Material

Additional file 1: Table S2 Proportion of CBABYJN7-4 mice resistant or susceptible to pneumococcal infection, grouped by their genotype at either SNP 4_80 or SNP 4_103. Click here for file

Additional file 2: Figure S1 Kaplan-Meier survival curves showing the cumulative survival for the CBABYJN7-4 mice, based on genotype at SNP 4_103. The survival curve of each group is labelled by genotype. Click here for file

Additional file 3: Figure S2 A) Kaplan-Meier survival curves showing the cumulative survival for the BYJCBAN6-4 mice, based on genotype at SNP 4_38. The survival curve of each group is labelled by genotype. B) Kaplan-Meier survival curves showing the cumulative survival for the BYJCBAN6-4 mice, based on genotype at SNP 4_103. The survival curve of each group is labelled by genotype. Click here for file

Additional file 4: Table S1 Panel of PCR and Pyrosequencing primers used to genotype SNPs in CCBAF2 mice. Click here for file
