# Supplementary material for: Spir2; a novel QTL on chromosome 4 contributes to susceptibility to pneumococcal infection in mice
Source: BMC Genomics. 2013 Apr 11;14:242. doi: 10.1186/1471-2164-14-242 (PMC3751763; doi:10.1186/1471-2164-14-242)
Supplement: Additional file 1: Table S2 — Proportion of CBABYJN7-4 mice resistant or susceptible to pneumococcal infection, grouped by their genotype at either SNP 4_80 or SNP 4_103. [file 1471-2164-14-242-S1.doc]

Supplementary Table 2. Proportion of CBABYJN7-4 mice resistant or susceptible to pneumococcal infection, grouped by their genotype at either SNP 4_80 or SNP 4_103

| **CBABYJN7-4** | **4-80** | | | **4-103** | | |
| --- | --- | --- | --- | --- | --- | --- |
|  | CBA/CaH | Heterozygous | BALB/cByJ | CBA/CaH | Heterozygous | BALB/cByJ |
| Total | 17 | 37 | 19 | 21 | 35 | 17 |
| Susceptible | 15/17 | 24/37 | 16/19 | 18/21 | 23/35 | 14/17 |
|  | (88%) | (65%) | (84%) | (86%) | (66%) | (82%) |
| Resistant | 2/17 | 13/37 | 3/19 | 3/21 | 12/35 | 3/17 |
|  | (12%) | (35%) | (16%) | (14%) | (34%) | (18%) |
