# Supplementary figures and images for: Spir2; a novel QTL on chromosome 4 contributes to susceptibility to pneumococcal infection in mice
Source: BMC Genomics. 2013 Apr 11;14:242. doi: 10.1186/1471-2164-14-242 (PMC3751763; doi:10.1186/1471-2164-14-242)

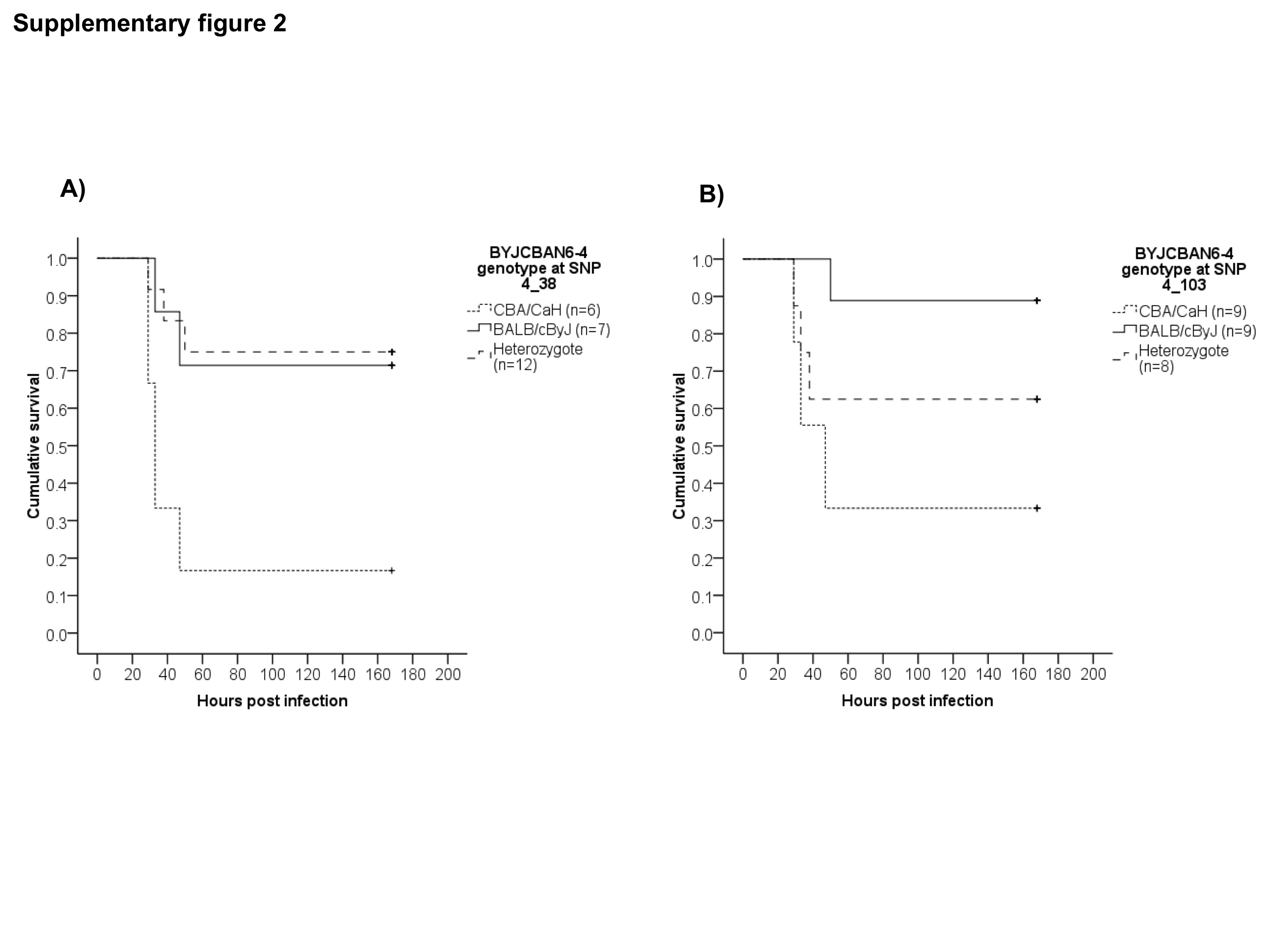

Supplement: Additional file 3: Figure S2 — A) Kaplan-Meier survival curves showing the cumulative survival for the BYJCBAN6-4 mice, based on genotype at SNP 4_38. The survival curve of each group is labelled by genotype. B) Kaplan-Meier survival curves showing the cumulative survival for the BYJCBAN6-4 mice, based on genotype at SNP 4_103. The survival curve of each group is labelled by genotype. [file 1471-2164-14-242-S3.jpeg]
