# Supplementary material for: Spir2; a novel QTL on chromosome 4 contributes to susceptibility to pneumococcal infection in mice
Source: BMC Genomics. 2013 Apr 11;14:242. doi: 10.1186/1471-2164-14-242 (PMC3751763; doi:10.1186/1471-2164-14-242)
Supplement: Additional file 4: Table S1 — Panel of PCR and Pyrosequencing primers used to genotype SNPs in CCBAF2 mice. [file 1471-2164-14-242-S4.doc]

**Supplementary Table 1**

| **Chr** | **SNP** | **Reference SNP / Celera ID** | **Forward primer** | **Reverse primer** | **Sequencing primer** | **Biotinylated primer** |
| --- | --- | --- | --- | --- | --- | --- |
| 1 | 1_29 | 01.029.481 | NNNGGGAGAGGAGGAAAGTGTGTGG | GCATACAGATGCCCAGGTTCAA | GAAAGTGTGTGGAAATTGT | R |
|  | 1_59 | rs3022796 | TGGACCAATGCTTAGAAGGGAGTC | ATGCGAGGATTTGGGGGAAT | TCTCCATGGTGCACA | F |
|  | 1_93 | rs3022829 | AGGACAGCAGCACCCATGC | TGACACCGAGTGACAGAGAAAGG | CCCTGGCCACTGTC | F |
|  | 1_128 | 01.128.089 | NNNGGATGTGGGCATTTTGTCGAA | TGTAATGCATGCCGCAACAA | CTTCTTTCTGCTTTTATTTT | R |
|  | 1_170 | rs3022851 | CACCAGATGTGTGCCTCTATGC | NNNGGGGGTTATGTTGTATGTGTCTG | TATGTTGTATGTGTCTGTAT | F |
| 2 | 2_38 | rs3022883 | AGGAACCCTGCCCTCCTTTG | GGTTTGGTGGGGTTGACCAG | ACAACTGAGAGCCTGG | F |
|  | 2_76 | 02.076.755 | NNNTCCTGTGGTTGAGGGGTCCT | GCAGTGACAGCAAATGAGTTCC | CCTCAACACTGACTGAACT | R |
|  | 2_104 | 02.101.477 | NNNGGATGCGAAGATGAGGCAAAC | GGGGAAAACCCATCAGCTTTG | CATTCTCTCCTCCAGC | R |
|  | 2_128.7 | rs13459166 | TTCGGAACGCAAAGTGGATG | NNNAAGCCTTTCCCCTGCCTCAC | CGGCTCACAAACCC | F |
|  | 2_181 | 02.181.947 | NNNGCAGGCATTGCTTTGTTCCA | GCGCGCCCAGATTACAGG | GAGAGGCCCTTCTGC | R |
| 3 | 3_37 | rs3022953 | CCCCATTAAAGCTGCCTTCTTCA | GGCTTCCCCCAGTGTGGAG | TGCCTTCTTCATTTTCTC | R |
|  | 3_81 | 03.081.771 | NNNTGAGGCTTTACAGTAGGTTGTGTTGC | TGTGCCAATAAGCCCATGGTA | GGTTGTGTTGCTAAACTATG | R |
|  | 3_105 | rs4224158 | CAAGAGGTGTGGTGGCATTTTTC | GCGCTCCTCCTCCAACTGAC | CTTTTTCACTTGGGACTT | F |
|  | 3_139 | 03.139.034 | NNNTTTCACCAAGGCAGAAGTGCAT | GGCGGAGTTTGGGAAGGATT | CCTACCTGTCTATGAACATC | R |
| 4 | 4_11 | 04.011.950 | NNNCCTGTCTTGCCCAGTCACACA | TGTGGAAAGACATGGCAAAAGC | ATCGTTTGAATGCCA | R |
|  | 4_38 | 04.034.828 | CCATCTGTGGCCCCTTCATC | TTTGATTTTATACATGACTTGGCACTT | CATGACTTGGCACTTTT | F |
|  | 4_47.7 | rs13477699 | TGGCCCCATAGCATTCACTT | NNNTGTCCCCATCCTGGAAGCTC | GCTTTGTGTATATCTGATCC | F |
|  | 4_75 | rs3704239 | NNNCCCCAGCAGAGACAGCAGAA | CCTGGGCTTGGGAATCTGAC | CTGGCCTGAGCTTTC | R |
|  | 4_80 | rs4224562 | CACGGACTGAAGCGAATGGA | NNNCTGCCCGGCTGATCACTCA | AGCCACTCACGGAAC | F |
|  | 4_87 | rs3022987 | TCAGGACCAAACAGCCCACA | NNNTCACCACCAAGACCATCAGGAA | GCCATTATGAGTCTTGAA | F |
|  | 4_103 | rs3662056 | NNNGGCTGCCTAGACCTGCATCC | TTTTCCATTCCTCATGCTCATTT | CCTAGACCTGCATCCA | R |
|  | 4_128 | 04.128.160 | CACTCCCCCCCATTAACCT | NNNCTCTGCCCTTGGTGATCAGTTC | ACTTGGTTCCTGGAGAT | F |
|  | 4_148 | 04.148.915 | NNNACCCGCCCGGCTTTTAGAG | GTGCTTGGGGTGGGACGATA | CTCAGGGGTCGCA | R |
| 5 | 5_23 | rs3023038 | TGGCCAACACTGGAAACAGC | NNNTCCCAGGAATGAAACAGATCCAA | CTTCCTGTTTTATTTGAGC | F |
|  | 5_51 | rs3023044 | CAAAGTACATTTGAGGACCTGTGAAA | NNNTCGAAGGTGGATGTTTGAGTCTG | GATGTTTGAGTCTGTAGGG | F |
|  | 5_76 | rs4225300 | NNNGGCAGGCCTGGTGACATTTT | GCAAATTTTGGCAACAATATAGAAA | GCAGATTTAGTGGATTGAG | R |
|  | 5_96 | 05.096.004 | NNNCACTCACCTGTGGCATCACAA | TGGTTCTTCCATGCTAACAGTGTCC | CCTGTGGCATCACAA | R |
|  | 5_137 | rs4225539 | NNNAGCCTTTGGTGATTGTGTGC | CTCACCCGCCTCCTTCTTGA | CCCGTCCCATGAAC | R |
| 6 | 6_22 | rs3088527 | NNNTGCCTCATTTGGAGTCTGGAT | TTCTGTCGGTTTCTGATCAAATGT | GGATATCTGTTGGTATATGG | R |
|  | 6_47.673 | 06.047.673 | NNNCACCCCTGCTCCTTCCATCA | TGTTTTCCTGTTTCCCACTGGTC | ATAGAAATACACAGCAGACA | R |
|  | 6_64 | 06.064.555 | NNNTGCACTGCCAAGCTTGATGA | GGTGGGTGGGTGGGTGTATG | TGCCAAGCTTGATGA | R |
|  | 6_87 | rs4226063 | TGGGGAGGCAAAAGAAAGCA | NNNCAGCAGAATCGGAAGGCGTTA | GAGGGTTAGAAGTTCAGATA | F |
|  | 6_115 | 06.115.250 | TTTGGCTGGCGAATGCATAA | NNNGCTCGTGTCTGTATCCAGGTCAT | GTCCTTTAATATTGTGT | F |
| 7 | 7_09 | rs3673010 | NNNTGTGTATGACTTATTTTCCCTTGTTCG | TGCACAACGTCCTGTGTTCC | CTTGTTCGATCATACTCTAA | R |
|  | 7_20 | Rs3655750 | TATAAAGATCTGGGGTGGC | GTCTCATTGAGCCGTAAGTGT | TGTACCCACATGGGTC | F |
|  | 7_23 | rs6168040 | GCTTTCAACCGATGTTAGAGATGC | NNNTGACTGGTGACATTTACAAAGTGCAG | GGTGTCTAACAGCACCC | F |
|  | 7_31 | Rs3147878 | TGAGGGGCAGAGAGAAAG | GAGTGGCACAGGTAACACA | GGTGGGGAGCCAGGA | R |
|  | 7_46 | Rs3089205 | CTCAGTGGAGGCTGTTTCT | GTCAGGAAACTGAGAGTCCA | CAGCAGTGGTGCCAT | F |
|  | 7_33 | rs3024058 | GGCACGGGGCTCTATCACTG | NNNTGCAACTACTCCAGAGGCTGAGG | CCAGAGGCTGAGGAA | F |
|  | 7_54 | rs4226656 | NNNCCATCTGCTACCAGCCATGAGA | TCTTTGTGAGCTTGACTTTGTCAGA | GCCATGAGAAATAAGAAAA | R |
|  | 7_72 | rs3023147 | CCCTGGACATGGCATCACCT | NNNCCCCTTCCGAGCACACAGAT | AAGCCAGGGCTGTG | F |
|  | 7_102 | rs3023155 | TCGCCATCTCCGCTTCTCTC | NNNCAGTGTCATGTGGACAGGAGAACA | ACAGAACACAATGACCAA | F |
|  | 7_123 | rs4228417 | NNNCCGTGCTCCCAAAGGTCTCA | TGGACATTCAATTGGGCCATA | CTTTCACAATGGGCA | R |
| 8 | 8_4 | 08.004.003 | TGACGTTATTGTTGTGATGACATTGC | NNNGAAACTCCATTTATTTGAGGTCATTGC | CATTGCAATGGTAACAAGT | F |
|  | 8_47 | 08.047.424 | GCCCACAGCAGCTGACCTTT | NNNCAACCAAACCATTCCAATGACG | AACCCACCTGCAACA | F |
|  | 8_77 | 08.077.729 | CCAACAGCAGCCCCAAACAT | NNNCCATGGTGTGTACAGTGCTTCCA | TGCTTCCAATTCTATTTGT | F |
|  | 8_95 | rs4227350 | NNNGCAGCTGAGGAGGAGCCAGA | GCAGCCAACGAGACCAGACA | CATCTTAAGCAAGGAG | R |
| 9 | 9_31.6 | rs13480146 | TCCCCTCTGCACAAAACTCCA | NNNGGCCCATGCTCCTAACCAGA | CATGCTCCTAACCAGAG | F |
|  | 9_57 | rs4227685 | NNNTGAGGGGTCATAGATGAGTGTTGC | ACAGGAGCAGGGACGACAGC | TGAGTGTTGCATATCACC | R |
|  | 9_73 | rs13480273 | NNNGCAGCTTCTGCATACACTCAGGA | CCCTGGAAAATTCCACAGCA | TTACCAGAAAGGTGGG | R |
|  | 9_97.4 | 09.097.417 | NNNTCTTTTGGGGAAGAGGACTGGA | CAAGCCTGCCATTTGAACCA | AGAATAATCCTTCATCCTG | R |
| 10 | 10_28 | rs3023233 | NNNACGGGGTGGGAGGCTTTTAC | TGGCATGAAATGTGGATGCTC | GGACTTGATCAGTTTCTGT | R |
|  | 10_56 | rs3089794 | NNNGCAGAAAGAAGGAGGAAGAAGAA | TTTGGGGCTTCATTTGCTTG | AAGAAGGAGGAAGAAGAA | R |
|  | 10_89 | rs3089366 | NNNTCCCACTCATCACCTTCATCTCC | TGTCCCGGCTTGGTAAATGG | TCATCTCCAAGTTCAAAAT | R |
|  | 10_105 | rs3088857 | AAAGGGAGGGGCTGAGATGG | NNNCCCAAACTGAAATCAAATTATACGG | AAATTCCTAGAAAGTT | F |
| 11 | 11_13 | 11.013.172 | NNNTGGTTCATTATGATGAGGGCATTT | CATGTGCTTTAATGTACTTGCAATCG | GCCCAAATAATTAGAGACAA | R |
|  | 11_54 | rs3023258 | NNNAATTTGGCAGTTCCTCCCATT | TCATGCACACATACAGGCATGG | CCCATTATTTTTTTTTCTGA | R |
|  | 11_94 | rs3089065 | NNNCAGTGAGATGGGAGAGACGTG | TGGTTGGCAATGGCTATTTTGA | ATAAATATTAAAAGGAGTGT | R |
| 12 | 12_12 | rs3090133 | NNNGGGTATCTCACAACCAAACCTTTCA | GGGGGAAGGACAGAGATGCT | ATCCTTTACATGAAAGACAC | R |
|  | 12_68 | rs3021895 | NNNTGGCAGCTCACAATGGTCTG | TTCACAGAACTCTCTTGCAACTTTTT | AGACATGCAAGCAAAAT | R |
|  | 12_100 | 12.100.101 | NNNTGATCCGGGATAAATTCTGAATAG | TGCCAAAGAAGCAGCCCTTA | TCTTTAAACATATCTTGACC | R |
| 13 | 13_17 | 13.017.126 | NNNGCCACCTGGGATGACTGCTAA | TGTGGTTTGCTTTAAACGGTCCT | CAACCAGCTCCTTGTT | R |
|  | 13_59 | rs3023383 | NNNTCCCTGGGGCTTATTGGCTA | TCAGGAGCCATCCACTTTGC | CTTCATCTGAGAGCCC | R |
|  | 13_93 | rs3023390 | NNNGGTTCTTTCCGTGCTGTGTTC | CAGAGCACCGGTACCATCCA | CGTGCTGTGTTCTGGA | R |
| 14 | 14_16 | rs4230209 | NNNTCTGTAGCGGCTTTGTTTTTATTT | CGGACCCGTGGTTCAAAAGT | ATTTATACTCTACAAGGGAT | R |
|  | 14_53 | 14.053.110 | NNNCCTGGTAAACCGAGGGTAAGGA | TGAGCCACAGGACCACTGGA | GATCCTAATCCCATAGAGA | R |
|  | 14_93.1 | 14.093.157a | NNNGCATCCTGGGACTTCTATGCAA | CGCAGAACTTTGCCATTGGTT | ATATAAAAAGCTAATGAAAT | R |
| 15 | 15_32 | rs3091174 | TGCTCCCCACCTAGACCTCCT | TTCTTTTAGTTTTCACAAAACGTTGG | CCATCCCGCTCATC | R |
|  | 15_52 | rs3023676 | GCCCAAGACCAACAGTAGGAGAGA | NNNCACCAATAATATCACAGGTCATCTGG | CATCTGGGGTCCTTT | F |
|  | 15_87 | 15.087.204 | GGTGTCTCTCCTTTCTCAGGACCA | NNNGTGGACTTGGCTGCATCTGG | CCCCAAGGCCATACTA | F |
| 16 | 16_10 | rs3023432 | NNNATGTGCTGCCAAAACGAGCA | TCCTGGCTGGCATCATTGAA | CCCTTGGAGACTGGA | R |
|  | 16_33 | rs4172185 | TCCAAGACAGCCAGGGGAAC | NNNCAAGGCCCAGTGGACATGGT | ACATGGTCCTAGCACTG | F |
|  | 16_61.8 | 16.061.878 | NNNTTTGGCCATCCACCCTTGTC | TGATGTATCTGGGAAAGCAGGTTC | TCCCTTCCTTCTACTTTG | R |
| 17 | 17_9 | 17.009.615 | NNNCTTCACCCCCAACCACCACT | CCGGCACGGGTGTAGTTCAG | AGCTGAGCCAGCG | R |
|  | 17_32M | rs3023442 | CGACCCGCTCCCTATTAGACG | GGAGGGTGGAGACCGGAGAT | GGGGTTTCAGGTACCTC | R |
|  | 17_71 | rs3023668 | NNNGGAAGGTTGAGGACCACTGC | TTGCTTGCTGGATGAATGAACA | GCAGAGGCACAATTAAGTA | R |
| 18 | 18_25.4 | 18.025.479 | NNNCAGATGGCTTCACGGCTTTG | CCACTTTCTCCCTGTTCCTTGTG | GCTTTGTCTTCTCTTTATAG | R |
|  | 18_62 | rs4231898 | GCAAGTTTCAAGTCCATGGCAAG | NNNGGGCAAATAGAGAGTTTCCAGAGC | CTAGTTATTTATTTCCACAT | F |
|  | 18_80 | rs4231968 | CCCTGCCTGTGGGTCAGACT | NNNGTTCTCCTGCCGCTGAGGTC | GCCTCCGTGAGTGACT | F |
| 19 | 19_19 | rs3023480 | CGGAAGAAAGTGGAGGACAATGA | NNNCCCTAAGGGAGATTACCTAAGAATTTG | TCTTAATGTCAATCCCAATA | F |
|  | 19_35 | 19.035.019 | NNNGCACGGGCTTGGAGTCACAT | TGGAAAGGTATAAATATCCCACGTGTT | TCAGATAAGGTGCCAAC | R |
|  | 19_55 | 19.055.858 | GGCTCATGGCGGAAGTGTCT | NNNATAAGCACCCACCGCCCATA | GCTGCAGCCCAGG | F |
